# Desvenlafaxinium chloranilate ethyl acetate solvate

**DOI:** 10.1107/S1600536813025312

**Published:** 2013-09-18

**Authors:** Manpreet Kaur, Jerry P. Jasinski, Ray J. Butcher, H. S. Yathirajan, K. Byrappa

**Affiliations:** a Department of Studies in Chemistry, University of Mysore, Manasagangotri, Mysore 570 006, India; b Department of Chemistry, Keene State College, 229 Main Street, Keene, NH 03435-2001, USA; c Department of Chemistry, Howard University, 525 College Street NW, Washington, DC 20059, USA; d Materials Science Center, University of Mysore, NCHS Building, Manasagangotri, Mysore 570 006, India

## Abstract

In the cation of the title compound, C_16_H_26_NO_2_^+^·C_6_HCl_2_O_4_^−^·C_4_H_8_O_2_, the 1-hy­droxy-cyclo­hexyl ring adopts a slightly distorted chair conformation. The dihedral angle between the mean planes of the 1-hy­droxy­cyclo­hexyl and 4-hy­droxy­phenyl rings is 84.0 (8)°. In the anion, the hydroxyl H atom is twisted slightly out of the ring plane with a C—C—O—H torsion angle of −171.9°. Disorder was modeled for the methyl group of the acetate group in the solvate with an occupancy ratio of 0.583 (15): 0.417 (15). In the crystal, O—H⋯O hydrogen bonds are observed between cations and between cations and anions, while bifuricated N—H⋯(O,O) cation–anion hydrogen bonds are also present, forming chains along [010] and [100]. In addition weak cation–anion and cation–solvate C—H⋯O inter­actions occur.

## Related literature

For the pharmacological importance of Desvenlafaxine {systematic name: 4-[2-di­methyl­amino-1-(1-hy­droxy­cyclo­hex­yl)eth­yl]phenol}, see: Deecher *et al.* (2006 ▶). For related structures, see: Dayananda *et al.* (2012 ▶); Duggirala *et al.* (2009 ▶); Hadfield *et al.* (2004 ▶); Mungkornasawakul *et al.* (2009 ▶); Sivalakshmidevi *et al.* (2002 ▶); Sun *et al.* (2006 ▶); Tessler & Goldberg (2004 ▶); Vega *et al.* (2000 ▶); Venu *et al.* (2008 ▶); Zhang *et al.* (2006 ▶). For puckering parameters, see: Cremer & Pople (1975 ▶). For standard bond lengths, see: Allen *et al.* (1987 ▶).
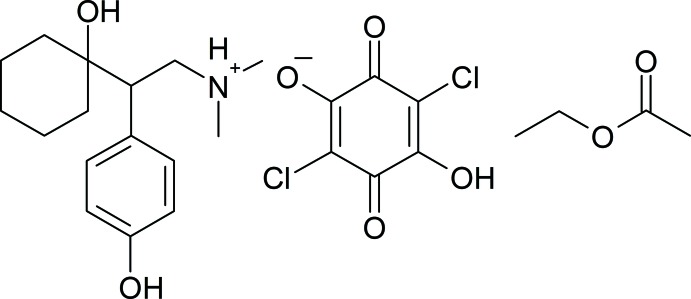


## Experimental

### 

#### Crystal data

C_16_H_26_NO_2_^+^·C_6_HCl_2_O_4_^−^·C_4_H_8_O_2_
*M* *_r_* = 560.45
Monoclinic, 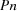

*a* = 11.1738 (2) Å
*b* = 9.39846 (16) Å
*c* = 13.6046 (2) Å
β = 105.109 (2)°
*V* = 1379.33 (4) Å^3^
*Z* = 2
Mo *K*α radiation
μ = 0.28 mm^−1^
*T* = 123 K
0.44 × 0.29 × 0.12 mm

#### Data collection

Agilent Xcalibur, Ruby, Gemini diffractometer
Absorption correction: multi-scan (*CrysAlis PRO* and *CrysAlis RED*; Agilent, 2012 ▶) *T*_min_ = 0.991, *T*_max_ = 1.000
16094 measured reflections
8667 independent reflections
7926 reflections with *I* > 2σ(*I*)
*R*_int_ = 0.024

#### Refinement

*R*[*F* ^2^ > 2σ(*F* ^2^)] = 0.057
*wR*(*F* ^2^) = 0.158
*S* = 1.05
8667 reflections
351 parameters
16 restraints
H atoms treated by a mixture of independent and constrained refinement
Δρ_max_ = 1.16 e Å^−3^
Δρ_min_ = −0.75 e Å^−3^
Absolute structure: Flack (1983 ▶), 3959 Friedel pairs
Absolute structure parameter: 0.01 (5)

### 

Data collection: *CrysAlis PRO* (Agilent, 2012 ▶); cell refinement: *CrysAlis PRO*; data reduction: *CrysAlis RED* (Agilent, 2012 ▶); program(s) used to solve structure: *SHELXS97* (Sheldrick, 2008 ▶); program(s) used to refine structure: *SHELXL97* (Sheldrick, 2008 ▶); molecular graphics: *SHELXTL* (Sheldrick, 2008 ▶); software used to prepare material for publication: *SHELXTL*.

## Supplementary Material

Crystal structure: contains datablock(s) I. DOI: 10.1107/S1600536813025312/hg5346sup1.cif

Structure factors: contains datablock(s) I. DOI: 10.1107/S1600536813025312/hg5346Isup2.hkl

Additional supplementary materials:  crystallographic information; 3D view; checkCIF report

## Figures and Tables

**Table 1:** Hydrogen-bond geometry (Å, °)

*D*—H⋯*A*	*D*—H	H⋯*A*	*D*⋯*A*	*D*—H⋯*A*
O1—H1⋯O2^i^	0.84	1.85	2.669 (2)	166
O2—H2⋯O4*A*	0.84	1.89	2.694 (3)	160
N1—H1*N*⋯O4*A*	0.89 (3)	1.95 (3)	2.775 (3)	153 (3)
N1—H1*N*⋯O3*A*	0.89 (3)	2.43 (3)	3.099 (3)	132 (2)
O2*A*—H2*A*1⋯O1^ii^	0.84	2.03	2.768 (3)	147
C3—H3*A*⋯O1*A* ^iii^	0.95	2.59	3.371 (3)	140
C9—H9*B*⋯O1*S*	0.98	2.29	3.224 (4)	159

Symmetry codes: (i) 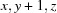; (ii) 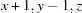; (iii) 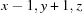.

**Fig. 1.**
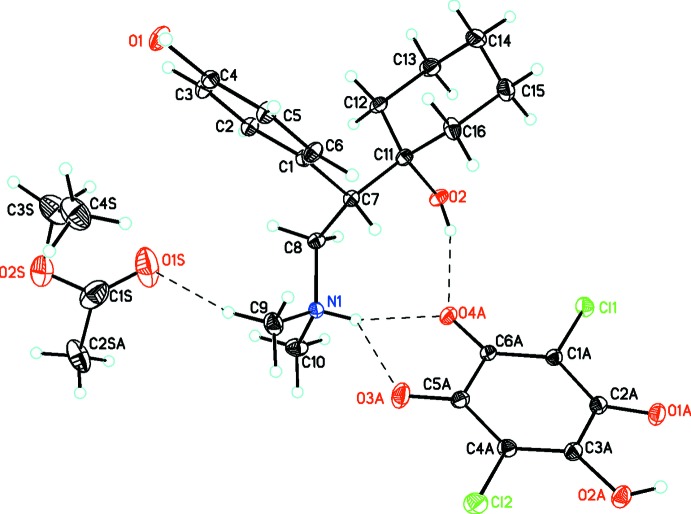
ORTEP drawings of cation (C16H26NO2+), anion (C6HCl2O4-), solvate (C4H8O2), in the asymmetric unit of (I) showing the atom labeling scheme and 30% probability displacement ellipsoids. Dashed lines indicate N—H···O, O—H···O hydrogen bonds and weak C—H···O intermolecular interactions. Only the major component of the disordered solvent methyl group is shown.

**Fig. 2.**
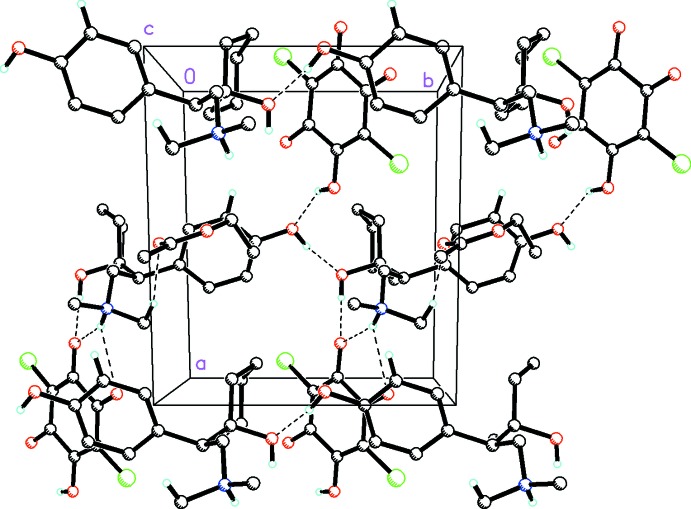
Molecular packing for (I) viewed along the c axis. Dashed lines indicate hydrogen bonds between cation (C16H26NO2+), anion (C6HCl2O4-) and solvate (C4H8O2) groups forming chains along [0 1 0] and [1 0 0]. Weak C—H···O cation-anion and cation-solvate intermolecular interactions also influence crystal packing. Only the major component of the disordered solvent methyl group is shown.

## supplementary crystallographic information

### 1. Comment

Desvenlafaxine {chemically, 4-[2-dimethylamino-1-(1-hydroxycyclohexyl)ethyl]phenol} also known as o-desmethylvenlafaxine, is an antidepressant of the serotonin-norepinephrine reuptake inhibitor class (Deecher *et al.*, 2006). Desvenlafaxine is a synthetic form of the major active metabolite of venlafaxine and is being targeted as the first non-hormonal based treatment for menopause. It is a racemic mixture and is reported to exist in four crystalline polymorphs (Hadfield *et al.*, 2004). The crystal structure of venlafaxine hydrochloride (Vega *et al.*, 2000), a monoclinic polymorph of venlafaxine hydrochloride (Sivalakshmidevi *et al.*, 2002), venlafaxine (Tessler & Goldberg, 2004), desvenlafaxine succinate monohydrate (Venu *et al.*, 2008) and two polytypes of desvenlafaxine succinate monohydrate (Duggirala *et al.*, 2009) have been reported. Some of the ethyl acetate solvate structures reported are: N-[(S)-1-(5-chloro-2-hydroxyphenyl)ethyl]-N-[(R)- 2-hydroxy-1-phenylethyl]aminium chloride ethyl acetate solvate (Zhang *et al.*, 2006), 5-ethoxycarbonyl-2,6-dimethyl-4-(3-nitrophenyl)-1,4-dihydropyridine- 3-carboxylic anhydride ethyl acetate solvate (Sun *et al.*, 2006) and stemofoline ethyl acetate solvate (Mungkornasawakul *et al.*, 2009). The crystal structure of triprolidinium dichloranilate-chloanilic acid-methanol-water is recently reported (Dayananda *et al.*, 2012).

In view of the importance of desvenlafaxine, this paper reports the crystal structure of the title solvate salt, (I), C_16_H_26_NO_2_^+^ . C_6_HCl_2_O_4_^-^ . C_4_H_8_O_2_.

The title compound. (I), crystallizes with one independent cation-pair and one solvate molecule in the asymmetric unit (Fig. 1). In the cation, the 1-hydroxy-cyclohexyl ring adopts a slightly distorted chair configuration with puckering parameters Q, θ, and φ = 0.569 (5)Å, 178.3 (4)° and 58.899 (1)°, respectively; (Cremer & Pople, 1975). Bond lengths are in normal ranges (Allen *et al.*, 1987). The dihedral angle between the mean planes of the 1-hydroxy-cyclohexyl and 4-hydroxy phenyl rings is 84.0 (8)°. In the anion, the hydroxyl H atom is slightly twisted with a C4A/C3A/O2A/H2A1 torsion angle of -171.9°. Disorder was modeled for the methyl carbon atom of the acetate group (C2SA) in the solvate with occupancies of 0.583 (15): 0.417 (15). In the crystal, O—H···O hydrogen bonds are observed between cations, cations and anions, (Table 1) while bifurcated N—H···O cation-anion hydrogen bonds are also present forming chains along [0 1 0] and [1 0 0], respectively (Fig. 2). In addition, weak cation-anion and cation-solvate C—H···O intermolecular interactions influence crystal packing.

### 2. Experimental

Desvenlafaxine succinate was obtained as a gift sample from R. L. Fine Chem, Bengaluru, India. A solution of desvenlafaxine succinate in water was treated with ammonium hydroxide solution till the pH > 7, a white precipitate of desvenlafaxine was obtained. The precipitate was filtered and dried overnight in open air and used as such for preparation of chloranilate salt. Desvenlafaxine (200 mg, 0.76 mmol) and chloranilic acid (159 mg, 0.76 mmol) were dissolved in hot methanol solution and stirred over a heating magnetic stirrer for 30 minutes at 333K . The resulting solution was allowed to cool slowly at room temperature. The salt formed was filtered & dried in vaccum desiccator over phosphorous pentoxide . The resulting compound was recrystallised from ethyl acetate solution yielding dark purple colored crystals (M.P.: 375 - 383 K).

### 3. Refinement

H1N was located by a difference map and refined isotropically. All of the remaining H atoms were placed in their calculated positions and then refined using the riding model with atom—H lengths of 0.95 or 1.00Å (CH), 0.99Å (CH_2_), 0.98Å (CH3) or 0.84Å (OH). Isotropic displacement parameters for these atoms were set to 1.2 (CH, CH_2_) or 1.5 (CH_3_, OH) times *U*_eq_ of the parent atom.

### Crystal data

**Table:**

C_16_H_26_NO_2_^+^·C_6_HCl_2_O_4_^−^·C_4_H_8_O_2_	*F*(000) = 592
*M**_r_* = 560.45	*D*_x_ = 1.349 Mg m^−^^3^
Monoclinic, *P**n*	Mo *K*α radiation, λ = 0.7107 Å
Hall symbol: P -2yac	Cell parameters from 8855 reflections
*a* = 11.1738 (2) Å	θ = 3.1–32.6°
*b* = 9.39846 (16) Å	µ = 0.28 mm^−^^1^
*c* = 13.6046 (2) Å	*T* = 123 K
β = 105.109 (2)°	Prism, black
*V* = 1379.33 (4) Å^3^	0.44 × 0.29 × 0.12 mm
*Z* = 2	

### Data collection

**Table:**

Agilent Xcalibur, Ruby, Gemini diffractometer	8667 independent reflections
Radiation source: Enhance (Mo) X-ray Source	7926 reflections with *I* > 2σ(*I*)
Graphite monochromator	*R*_int_ = 0.024
Detector resolution: 10.5081 pixels mm^-1^	θ_max_ = 32.7°, θ_min_ = 3.1°
ω scans	*h* = −16→16
Absorption correction: multi-scan (*CrysAlis PRO* and *CrysAlis RED*; Agilent, 2012)	*k* = −12→13
*T*_min_ = 0.991, *T*_max_ = 1.000	*l* = −19→20
16094 measured reflections	

### Refinement

**Table:**

Refinement on *F*^2^	Secondary atom site location: difference Fourier map
Least-squares matrix: full	Hydrogen site location: inferred from neighbouring sites
*R*[*F*^2^ > 2σ(*F*^2^)] = 0.057	H atoms treated by a mixture of independent and constrained refinement
*wR*(*F*^2^) = 0.158	*w* = 1/[σ^2^(*F*_o_^2^) + (0.0965*P*)^2^ + 0.5142*P*] where *P* = (*F*_o_^2^ + 2*F*_c_^2^)/3
*S* = 1.05	(Δ/σ)_max_ = 0.006
8667 reflections	Δρ_max_ = 1.16 e Å^−^^3^
351 parameters	Δρ_min_ = −0.75 e Å^−^^3^
16 restraints	Absolute structure: Flack (1983), ???? Friedel pairs
Primary atom site location: structure-invariant direct methods	Absolute structure parameter: 0.01 (5)

### Special details

**Table:**

Geometry. All esds (except the esd in the dihedral angle between two l.s. planes) are estimated using the full covariance matrix. The cell esds are taken into account individually in the estimation of esds in distances, angles and torsion angles; correlations between esds in cell parameters are only used when they are defined by crystal symmetry. An approximate (isotropic) treatment of cell esds is used for estimating esds involving l.s. planes.
Refinement. Refinement of F^2^ against ALL reflections. The weighted R-factor wR and goodness of fit S are based on F^2^, conventional R-factors R are based on F, with F set to zero for negative F^2^. The threshold expression of F^2^ > 2sigma(F^2^) is used only for calculating R-factors(gt) etc. and is not relevant to the choice of reflections for refinement. R-factors based on F^2^ are statistically about twice as large as those based on F, and R- factors based on ALL data will be even larger.

### Fractional atomic coordinates and isotropic or equivalent isotropic displacement parameters (Å^2^)

**Table:**

	*x*	*y*	*z*	*U*_iso_*/*U*_eq_	Occ. (<1)
O1	0.47746 (18)	1.43604 (18)	0.31751 (16)	0.0265 (4)	
H1	0.5338	1.4903	0.3093	0.040*	
O2	0.62810 (17)	0.62374 (17)	0.26371 (15)	0.0223 (3)	
H2	0.7043	0.6213	0.2928	0.033*	
N1	0.73387 (19)	0.7670 (2)	0.52239 (15)	0.0185 (3)	
H1N	0.792 (3)	0.737 (3)	0.493 (2)	0.012 (6)*	
C1	0.5942 (2)	1.0116 (2)	0.33682 (17)	0.0178 (4)	
C2	0.4800 (2)	1.0512 (2)	0.35322 (18)	0.0197 (4)	
H2A	0.4279	0.9801	0.3696	0.024*	
C3	0.4413 (2)	1.1925 (2)	0.34606 (19)	0.0214 (4)	
H3A	0.3633	1.2171	0.3570	0.026*	
C4	0.5178 (2)	1.2981 (2)	0.32262 (17)	0.0192 (4)	
C5	0.6322 (2)	1.2609 (2)	0.3069 (2)	0.0235 (4)	
H5A	0.6847	1.3320	0.2911	0.028*	
C6	0.6692 (2)	1.1186 (2)	0.31463 (19)	0.0216 (4)	
H6A	0.7476	1.0942	0.3045	0.026*	
C7	0.6381 (2)	0.8573 (2)	0.34508 (17)	0.0171 (3)	
H7A	0.7302	0.8614	0.3567	0.020*	
C8	0.6151 (2)	0.7795 (2)	0.43892 (17)	0.0194 (4)	
H8A	0.5530	0.8329	0.4646	0.023*	
H8B	0.5815	0.6834	0.4187	0.023*	
C9	0.7782 (3)	0.9077 (3)	0.5675 (2)	0.0312 (5)	
H9A	0.8571	0.8957	0.6191	0.047*	
H9B	0.7166	0.9485	0.5993	0.047*	
H9C	0.7899	0.9717	0.5139	0.047*	
C10	0.7170 (3)	0.6668 (3)	0.6020 (2)	0.0311 (5)	
H10A	0.7952	0.6574	0.6548	0.047*	
H10B	0.6919	0.5735	0.5713	0.047*	
H10C	0.6527	0.7033	0.6325	0.047*	
C11	0.5888 (2)	0.7699 (2)	0.24491 (18)	0.0195 (4)	
C12	0.4476 (2)	0.7658 (3)	0.20590 (19)	0.0229 (4)	
H12A	0.4154	0.8644	0.1965	0.027*	
H12B	0.4125	0.7193	0.2574	0.027*	
C13	0.4050 (3)	0.6854 (3)	0.1051 (2)	0.0345 (6)	
H13A	0.4278	0.5838	0.1163	0.041*	
H13B	0.3136	0.6914	0.0802	0.041*	
C14	0.4635 (3)	0.7462 (4)	0.0246 (2)	0.0412 (7)	
H14A	0.4379	0.6884	−0.0382	0.049*	
H14B	0.4336	0.8446	0.0079	0.049*	
C15	0.6038 (3)	0.7465 (3)	0.0623 (2)	0.0356 (6)	
H15A	0.6397	0.7905	0.0103	0.043*	
H15B	0.6344	0.6474	0.0732	0.043*	
C16	0.6452 (3)	0.8295 (3)	0.1618 (2)	0.0271 (5)	
H16A	0.6205	0.9303	0.1491	0.033*	
H16B	0.7368	0.8260	0.1862	0.033*	
Cl1	0.92007 (8)	0.40363 (7)	0.25545 (6)	0.02721 (13)	
Cl2	1.28410 (9)	0.82486 (7)	0.55657 (6)	0.03231 (14)	
O1A	1.19739 (19)	0.4153 (2)	0.31197 (16)	0.0300 (4)	
O2A	1.34999 (18)	0.5944 (2)	0.42885 (16)	0.0284 (4)	
H2A1	1.3584	0.5362	0.3842	0.043*	
O3A	1.0108 (2)	0.7925 (3)	0.52380 (19)	0.0397 (5)	
O4A	0.85246 (17)	0.6185 (2)	0.39869 (15)	0.0254 (3)	
C1A	1.0159 (2)	0.5140 (2)	0.34403 (17)	0.0197 (4)	
C2A	1.1458 (2)	0.5020 (3)	0.35560 (18)	0.0205 (4)	
C3A	1.2306 (2)	0.6058 (2)	0.42625 (18)	0.0212 (4)	
C4A	1.1866 (2)	0.7035 (2)	0.48058 (19)	0.0221 (4)	
C5A	1.0544 (2)	0.7100 (3)	0.47477 (19)	0.0233 (4)	
C6A	0.9653 (2)	0.6072 (2)	0.40123 (17)	0.0186 (4)	
O1S	0.5308 (4)	0.9814 (5)	0.6374 (3)	0.0818 (12)	
O2S	0.4947 (3)	1.1593 (3)	0.7334 (2)	0.0530 (7)	
C1S	0.5324 (4)	1.0314 (5)	0.7221 (4)	0.0620 (11)	
C2SA	0.5724 (11)	0.9564 (8)	0.8264 (4)	0.0518 (16)	0.583 (15)
H2S1	0.5631	0.8533	0.8167	0.078*	0.583 (15)
H2S2	0.5202	0.9892	0.8697	0.078*	0.583 (15)
H2S3	0.6593	0.9791	0.8591	0.078*	0.583 (15)
C2SB	0.6165 (11)	0.9595 (13)	0.8173 (7)	0.0518 (16)	0.417 (15)
H2S4	0.6065	0.8560	0.8112	0.078*	0.417 (15)
H2S5	0.5932	0.9922	0.8781	0.078*	0.417 (15)
H2S6	0.7031	0.9845	0.8229	0.078*	0.417 (15)
C3S	0.4586 (5)	1.2355 (5)	0.6368 (3)	0.0573 (10)	
H3SA	0.4192	1.1665	0.5831	0.069*	
H3SB	0.3946	1.3063	0.6412	0.069*	
C4S	0.5560 (5)	1.3091 (5)	0.6040 (3)	0.0588 (11)	
H4SA	0.5193	1.3843	0.5556	0.088*	
H4SB	0.5997	1.2412	0.5710	0.088*	
H4SC	0.6147	1.3512	0.6632	0.088*	

### Atomic displacement parameters (Å^2^)

**Table:**

	*U*^11^	*U*^22^	*U*^33^	*U*^12^	*U*^13^	*U*^23^
O1	0.0254 (8)	0.0144 (7)	0.0431 (10)	0.0010 (6)	0.0148 (8)	0.0025 (7)
O2	0.0191 (7)	0.0141 (7)	0.0317 (8)	0.0010 (5)	0.0029 (6)	0.0004 (6)
N1	0.0200 (8)	0.0184 (8)	0.0176 (7)	0.0013 (6)	0.0055 (7)	−0.0005 (6)
C1	0.0201 (9)	0.0148 (8)	0.0189 (9)	0.0006 (7)	0.0057 (7)	0.0016 (7)
C2	0.0202 (9)	0.0156 (9)	0.0251 (10)	−0.0014 (7)	0.0089 (8)	0.0022 (8)
C3	0.0198 (9)	0.0175 (9)	0.0283 (11)	−0.0009 (7)	0.0087 (9)	−0.0017 (8)
C4	0.0223 (9)	0.0150 (9)	0.0203 (9)	−0.0010 (7)	0.0058 (8)	0.0008 (7)
C5	0.0232 (10)	0.0151 (9)	0.0345 (12)	−0.0018 (7)	0.0117 (9)	0.0029 (8)
C6	0.0192 (9)	0.0165 (9)	0.0309 (11)	0.0012 (7)	0.0098 (8)	0.0030 (8)
C7	0.0173 (8)	0.0147 (8)	0.0199 (9)	−0.0005 (6)	0.0061 (7)	0.0018 (7)
C8	0.0180 (9)	0.0186 (9)	0.0216 (9)	−0.0002 (7)	0.0052 (8)	0.0042 (7)
C9	0.0332 (12)	0.0308 (12)	0.0292 (12)	−0.0045 (10)	0.0076 (10)	−0.0100 (10)
C10	0.0335 (12)	0.0331 (13)	0.0258 (11)	0.0007 (10)	0.0063 (10)	0.0117 (10)
C11	0.0211 (9)	0.0138 (8)	0.0235 (10)	0.0017 (7)	0.0056 (8)	0.0011 (7)
C12	0.0200 (9)	0.0204 (10)	0.0263 (10)	0.0043 (8)	0.0026 (8)	0.0004 (8)
C13	0.0324 (13)	0.0308 (13)	0.0324 (13)	0.0056 (10)	−0.0056 (11)	−0.0094 (10)
C14	0.0528 (18)	0.0419 (16)	0.0235 (12)	0.0184 (14)	0.0002 (12)	−0.0048 (11)
C15	0.0486 (17)	0.0328 (14)	0.0275 (12)	0.0094 (11)	0.0135 (12)	−0.0048 (10)
C16	0.0362 (13)	0.0239 (11)	0.0250 (10)	−0.0016 (9)	0.0149 (10)	−0.0010 (9)
Cl1	0.0233 (2)	0.0315 (3)	0.0264 (2)	−0.0068 (2)	0.0058 (2)	−0.0084 (2)
Cl2	0.0268 (3)	0.0285 (3)	0.0361 (3)	−0.0017 (2)	−0.0016 (2)	−0.0107 (3)
O1A	0.0235 (8)	0.0338 (10)	0.0336 (10)	0.0004 (7)	0.0091 (8)	−0.0118 (8)
O2A	0.0199 (8)	0.0311 (9)	0.0350 (10)	−0.0031 (6)	0.0087 (7)	−0.0084 (8)
O3A	0.0288 (10)	0.0425 (12)	0.0469 (13)	0.0034 (8)	0.0086 (9)	−0.0233 (10)
O4A	0.0179 (7)	0.0300 (9)	0.0278 (8)	0.0035 (6)	0.0050 (6)	−0.0038 (7)
C1A	0.0184 (9)	0.0204 (9)	0.0202 (9)	0.0000 (7)	0.0046 (8)	−0.0005 (7)
C2A	0.0186 (9)	0.0215 (10)	0.0219 (10)	−0.0022 (7)	0.0061 (8)	−0.0007 (8)
C3A	0.0199 (10)	0.0214 (10)	0.0225 (10)	0.0001 (7)	0.0059 (8)	0.0010 (8)
C4A	0.0224 (10)	0.0192 (9)	0.0221 (10)	−0.0005 (8)	0.0013 (8)	−0.0037 (8)
C5A	0.0204 (10)	0.0240 (10)	0.0234 (10)	0.0037 (8)	0.0019 (8)	−0.0023 (8)
C6A	0.0178 (9)	0.0191 (9)	0.0185 (9)	0.0029 (7)	0.0039 (7)	0.0015 (7)
O1S	0.064 (2)	0.100 (3)	0.090 (2)	−0.025 (2)	0.0364 (19)	−0.057 (2)
O2S	0.0581 (16)	0.0435 (14)	0.0621 (17)	−0.0014 (12)	0.0242 (13)	−0.0122 (13)
C1S	0.0401 (19)	0.049 (2)	0.091 (3)	0.0023 (16)	0.007 (2)	−0.009 (2)
C2SA	0.090 (5)	0.040 (2)	0.036 (2)	−0.024 (3)	0.036 (3)	−0.0036 (17)
C2SB	0.090 (5)	0.040 (2)	0.036 (2)	−0.024 (3)	0.036 (3)	−0.0036 (17)
C3S	0.061 (2)	0.067 (3)	0.0426 (19)	−0.022 (2)	0.0122 (18)	−0.0046 (18)
C4S	0.081 (3)	0.047 (2)	0.047 (2)	−0.021 (2)	0.014 (2)	−0.0082 (16)

### Geometric parameters (Å, º)

**Table:**

O1—C4	1.368 (3)	C14—C15	1.517 (5)
O1—H1	0.8400	C14—H14A	0.9900
O2—C11	1.444 (3)	C14—H14B	0.9900
O2—H2	0.8400	C15—C16	1.526 (4)
N1—C10	1.484 (3)	C15—H15A	0.9900
N1—C9	1.487 (3)	C15—H15B	0.9900
N1—C8	1.510 (3)	C16—H16A	0.9900
N1—H1N	0.89 (3)	C16—H16B	0.9900
C1—C6	1.392 (3)	Cl1—C1A	1.733 (2)
C1—C2	1.402 (3)	Cl2—C4A	1.723 (2)
C1—C7	1.526 (3)	O1A—C2A	1.236 (3)
C2—C3	1.392 (3)	O2A—C3A	1.330 (3)
C2—H2A	0.9500	O2A—H2A1	0.8400
C3—C4	1.401 (3)	O3A—C5A	1.205 (3)
C3—H3A	0.9500	O4A—C6A	1.256 (3)
C4—C5	1.395 (3)	C1A—C6A	1.386 (3)
C5—C6	1.395 (3)	C1A—C2A	1.423 (3)
C5—H5A	0.9500	C2A—C3A	1.516 (3)
C6—H6A	0.9500	C3A—C4A	1.348 (3)
C7—C8	1.550 (3)	C4A—C5A	1.460 (3)
C7—C11	1.563 (3)	C5A—C6A	1.551 (3)
C7—H7A	1.0000	O1S—C1S	1.240 (6)
C8—H8A	0.9900	O2S—C1S	1.295 (5)
C8—H8B	0.9900	O2S—C3S	1.459 (6)
C9—H9A	0.9800	C1S—C2SA	1.543 (7)
C9—H9B	0.9800	C1S—C2SB	1.544 (7)
C9—H9C	0.9800	C2SA—H2S1	0.9800
C10—H10A	0.9800	C2SA—H2S2	0.9800
C10—H10B	0.9800	C2SA—H2S3	0.9800
C10—H10C	0.9800	C2SB—H2S4	0.9800
C11—C12	1.528 (3)	C2SB—H2S5	0.9800
C11—C16	1.536 (3)	C2SB—H2S6	0.9800
C12—C13	1.530 (4)	C3S—C4S	1.455 (6)
C12—H12A	0.9900	C3S—H3SA	0.9900
C12—H12B	0.9900	C3S—H3SB	0.9900
C13—C14	1.525 (5)	C4S—H4SA	0.9800
C13—H13A	0.9900	C4S—H4SB	0.9800
C13—H13B	0.9900	C4S—H4SC	0.9800
			
C4—O1—H1	109.5	H13A—C13—H13B	108.0
C11—O2—H2	109.5	C15—C14—C13	110.9 (2)
C10—N1—C9	110.8 (2)	C15—C14—H14A	109.5
C10—N1—C8	110.14 (19)	C13—C14—H14A	109.5
C9—N1—C8	112.0 (2)	C15—C14—H14B	109.5
C10—N1—H1N	111.7 (19)	C13—C14—H14B	109.5
C9—N1—H1N	105.4 (19)	H14A—C14—H14B	108.0
C8—N1—H1N	107 (2)	C14—C15—C16	110.4 (2)
C6—C1—C2	117.82 (19)	C14—C15—H15A	109.6
C6—C1—C7	120.20 (18)	C16—C15—H15A	109.6
C2—C1—C7	121.96 (18)	C14—C15—H15B	109.6
C3—C2—C1	121.46 (19)	C16—C15—H15B	109.6
C3—C2—H2A	119.3	H15A—C15—H15B	108.1
C1—C2—H2A	119.3	C15—C16—C11	112.3 (2)
C2—C3—C4	119.7 (2)	C15—C16—H16A	109.1
C2—C3—H3A	120.2	C11—C16—H16A	109.1
C4—C3—H3A	120.2	C15—C16—H16B	109.1
O1—C4—C5	122.22 (19)	C11—C16—H16B	109.1
O1—C4—C3	118.08 (19)	H16A—C16—H16B	107.9
C5—C4—C3	119.7 (2)	C3A—O2A—H2A1	109.5
C4—C5—C6	119.63 (19)	C6A—C1A—C2A	122.8 (2)
C4—C5—H5A	120.2	C6A—C1A—Cl1	120.00 (16)
C6—C5—H5A	120.2	C2A—C1A—Cl1	117.20 (17)
C1—C6—C5	121.7 (2)	O1A—C2A—C1A	126.1 (2)
C1—C6—H6A	119.1	O1A—C2A—C3A	115.9 (2)
C5—C6—H6A	119.1	C1A—C2A—C3A	118.0 (2)
C1—C7—C8	112.93 (17)	O2A—C3A—C4A	123.1 (2)
C1—C7—C11	113.69 (18)	O2A—C3A—C2A	114.93 (19)
C8—C7—C11	111.97 (17)	C4A—C3A—C2A	121.9 (2)
C1—C7—H7A	105.8	C3A—C4A—C5A	120.5 (2)
C8—C7—H7A	105.8	C3A—C4A—Cl2	121.06 (18)
C11—C7—H7A	105.8	C5A—C4A—Cl2	118.47 (17)
N1—C8—C7	110.75 (17)	O3A—C5A—C4A	123.1 (2)
N1—C8—H8A	109.5	O3A—C5A—C6A	118.3 (2)
C7—C8—H8A	109.5	C4A—C5A—C6A	118.60 (19)
N1—C8—H8B	109.5	O4A—C6A—C1A	126.2 (2)
C7—C8—H8B	109.5	O4A—C6A—C5A	115.83 (19)
H8A—C8—H8B	108.1	C1A—C6A—C5A	117.92 (19)
N1—C9—H9A	109.5	C1S—O2S—C3S	111.7 (4)
N1—C9—H9B	109.5	O1S—C1S—O2S	122.4 (5)
H9A—C9—H9B	109.5	O1S—C1S—C2SA	127.7 (5)
N1—C9—H9C	109.5	O2S—C1S—C2SA	109.8 (5)
H9A—C9—H9C	109.5	O1S—C1S—C2SB	118.1 (6)
H9B—C9—H9C	109.5	O2S—C1S—C2SB	116.9 (6)
N1—C10—H10A	109.5	C2SA—C1S—C2SB	20.1 (4)
N1—C10—H10B	109.5	C1S—C2SA—H2S1	109.5
H10A—C10—H10B	109.5	C1S—C2SA—H2S2	109.5
N1—C10—H10C	109.5	C1S—C2SA—H2S3	109.5
H10A—C10—H10C	109.5	C1S—C2SB—H2S4	109.5
H10B—C10—H10C	109.5	C1S—C2SB—H2S5	109.5
O2—C11—C12	106.03 (18)	H2S4—C2SB—H2S5	109.5
O2—C11—C16	108.25 (18)	C1S—C2SB—H2S6	109.5
C12—C11—C16	109.8 (2)	H2S4—C2SB—H2S6	109.5
O2—C11—C7	108.88 (18)	H2S5—C2SB—H2S6	109.5
C12—C11—C7	114.40 (17)	C4S—C3S—O2S	117.2 (4)
C16—C11—C7	109.33 (18)	C4S—C3S—H3SA	108.0
C11—C12—C13	112.1 (2)	O2S—C3S—H3SA	108.0
C11—C12—H12A	109.2	C4S—C3S—H3SB	108.0
C13—C12—H12A	109.2	O2S—C3S—H3SB	108.0
C11—C12—H12B	109.2	H3SA—C3S—H3SB	107.2
C13—C12—H12B	109.2	C3S—C4S—H4SA	109.5
H12A—C12—H12B	107.9	C3S—C4S—H4SB	109.5
C14—C13—C12	111.5 (2)	H4SA—C4S—H4SB	109.5
C14—C13—H13A	109.3	C3S—C4S—H4SC	109.5
C12—C13—H13A	109.3	H4SA—C4S—H4SC	109.5
C14—C13—H13B	109.3	H4SB—C4S—H4SC	109.5
C12—C13—H13B	109.3		
			
C6—C1—C2—C3	1.0 (3)	O2—C11—C16—C15	60.0 (3)
C7—C1—C2—C3	179.6 (2)	C12—C11—C16—C15	−55.3 (3)
C1—C2—C3—C4	−0.4 (4)	C7—C11—C16—C15	178.5 (2)
C2—C3—C4—O1	−179.2 (2)	C6A—C1A—C2A—O1A	175.1 (2)
C2—C3—C4—C5	−0.2 (3)	Cl1—C1A—C2A—O1A	−3.8 (3)
O1—C4—C5—C6	179.0 (2)	C6A—C1A—C2A—C3A	−6.0 (3)
C3—C4—C5—C6	0.1 (4)	Cl1—C1A—C2A—C3A	175.06 (16)
C2—C1—C6—C5	−1.1 (4)	O1A—C2A—C3A—O2A	2.9 (3)
C7—C1—C6—C5	−179.7 (2)	C1A—C2A—C3A—O2A	−176.1 (2)
C4—C5—C6—C1	0.6 (4)	O1A—C2A—C3A—C4A	−178.2 (2)
C6—C1—C7—C8	134.9 (2)	C1A—C2A—C3A—C4A	2.8 (3)
C2—C1—C7—C8	−43.6 (3)	O2A—C3A—C4A—C5A	−179.7 (2)
C6—C1—C7—C11	−96.1 (2)	C2A—C3A—C4A—C5A	1.5 (4)
C2—C1—C7—C11	85.4 (2)	O2A—C3A—C4A—Cl2	1.4 (3)
C10—N1—C8—C7	−167.94 (19)	C2A—C3A—C4A—Cl2	−177.43 (18)
C9—N1—C8—C7	68.3 (2)	C3A—C4A—C5A—O3A	177.9 (3)
C1—C7—C8—N1	−103.0 (2)	Cl2—C4A—C5A—O3A	−3.1 (4)
C11—C7—C8—N1	127.10 (19)	C3A—C4A—C5A—C6A	−2.8 (3)
C1—C7—C11—O2	−175.68 (16)	Cl2—C4A—C5A—C6A	176.18 (17)
C8—C7—C11—O2	−46.2 (2)	C2A—C1A—C6A—O4A	−176.3 (2)
C1—C7—C11—C12	−57.3 (2)	Cl1—C1A—C6A—O4A	2.6 (3)
C8—C7—C11—C12	72.2 (2)	C2A—C1A—C6A—C5A	4.7 (3)
C1—C7—C11—C16	66.2 (2)	Cl1—C1A—C6A—C5A	−176.40 (16)
C8—C7—C11—C16	−164.28 (18)	O3A—C5A—C6A—O4A	0.0 (4)
O2—C11—C12—C13	−62.9 (3)	C4A—C5A—C6A—O4A	−179.4 (2)
C16—C11—C12—C13	53.8 (3)	O3A—C5A—C6A—C1A	179.1 (2)
C7—C11—C12—C13	177.1 (2)	C4A—C5A—C6A—C1A	−0.3 (3)
C11—C12—C13—C14	−54.9 (3)	C3S—O2S—C1S—O1S	−3.2 (6)
C12—C13—C14—C15	55.8 (3)	C3S—O2S—C1S—C2SA	178.6 (5)
C13—C14—C15—C16	−56.6 (3)	C3S—O2S—C1S—C2SB	158.1 (7)
C14—C15—C16—C11	57.2 (3)	C1S—O2S—C3S—C4S	−86.0 (5)

### Hydrogen-bond geometry (Å, º)

**Table:**

*D*—H···*A*	*D*—H	H···*A*	*D*···*A*	*D*—H···*A*
O1—H1···O2^i^	0.84	1.85	2.669 (2)	166
O2—H2···O4*A*	0.84	1.89	2.694 (3)	160
N1—H1*N*···O4*A*	0.89 (3)	1.95 (3)	2.775 (3)	153 (3)
N1—H1*N*···O3*A*	0.89 (3)	2.43 (3)	3.099 (3)	132 (2)
O2*A*—H2*A*1···O1^ii^	0.84	2.03	2.768 (3)	147
C3—H3*A*···O1*A*^iii^	0.95	2.59	3.371 (3)	140
C9—H9*B*···O1*S*	0.98	2.29	3.224 (4)	159

Symmetry codes: (i) *x*, *y*+1, *z*; (ii) *x*+1, *y*−1, *z*; (iii) *x*−1, *y*+1, *z*.
